# Supplementary material for: BioREx: Improving Biomedical Relation Extraction by Leveraging Heterogeneous Datasets
Source: ArXiv. 2023 Jun 19:arXiv:2306.11189v1. Preprint. [Version 1] (PMC10370213)
Supplement: Supplement 1 [file NIHPP2306.11189v1-supplement-1.pdf]

Supplementary Material

Experiment A: The performance of BioREx using partial BioRED data

Because it is time-consuming and costly to create a multi-relation dataset, such as BioRED, the results of the previous experiments suggest that using our approach may effectively decrease the need for manual data annotation.

We conducted an experiment using different BioRED subsets to determine whether leveraging external datasets can achieve a similar (or better) performance compared to the original data. Specifically, we randomly sampled four subsets of the BioRED training data of different sizes for model development and evaluation on the independent BioRED test set. The detailed results are shown in Figure 1. With the eight external datasets, the performance of the models trained on five incremental sizes (100, 200, 300, 400, and 500 abstracts) of training data is improved significantly. In particular, the performance of the model trained on 60% of the training set (300 abstracts) achieved a better result (75.8%) than did the model trained on the entire training set of the original BioRED (74.4%). These results demonstrate that our approach can take advantage of external datasets to build reliable training data with fewer annotation efforts.

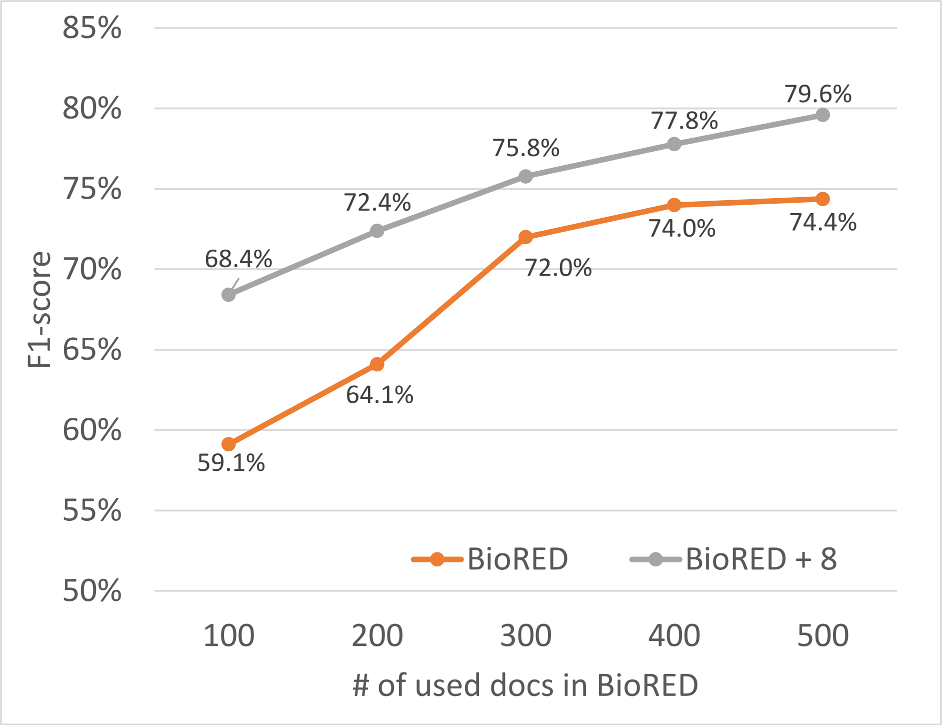

Figure 1. Performance of BioREx using partial BioRED with additional eight datasets.

We further compared the performance and efficiency of different PLM models. We chose five well-known pre-trained models for the comparison, including PubMedBERT [1], BioELECTRA [2], Bioformer [3], BioBert [4] and Roberta [5]. As shown in Table 1, we evaluated the final performance using the merged training set of BioRED with the eight corpora. PubMedBERT achieved the best performance, but Bioformer presents much more efficiency than do other models with close performance, as it is about two times faster on training and processing steps. Even though PubMedBERT achieved slightly higher performance than did Bioformer, Bioformer is more efficient than is PubMedBERT, which is an impressive advantage for processing large-scale data (e.g., entire PubMed abstracts, PMC full-text versions).

Table 1. Comparison of the PLM models

PLM	Precision	Recall	F-score	Training time (per epoch)	Processing time per 1000 pairs	
					GPU	CPU
PubMedBERT [1]	80.0	**79.2**	**79.6**	58m	100s	5.9m
BioELECTRA [2]	81.2	76.4	78.7	1h4m	90s	5.4m
Bioformer [3]	80.3	78.1	79.2	35m	55s	3.2m
BioBert [4]	**81.6**	69.1	74.8	1h4m	90s	5.9m
Roberta [5]	77.6	70.9	74.1	1h2m	87s	5.7m

Table S1. Existing relation corpora and the available relations. The event detection corpora (e.g., BioNLP share task) are not listed below. G: Gene/Protein/Variant, C: Chemical/Drug, and D: Disease/Phenotype.

Dataset	# Doc./Sent.	SEN/DOC	Relation					Not fully annotated (¥)<br>Specific scope (†)<br>Span is not provided (‡)<br>Not available (×)
			<G,G>	<G,C>	<C,C>	<G,D>	<C,D>	
BioRED [6]	600 abstracts	Document	●	●	●	●	●	
AIMed[7]	230 abstracts	Sentence	●					
HPRD50[8]	50 abstracts	Sentence	●					
DrugProt [9] & ChemProt [10]	5,000 abstracts	Sentence		●				
DDI [11]	905 abstracts	Sentence			●			
RENET2 [12]	500 full texts	Document	●					¥ Not fully annotated
RENET [13]	30,192 abstracts	Document	●					¥ Not fully annotated
EU-ADR [14]	300 abstracts	Sentence		●		●	●	¥ Not fully annotated
N-ary dataset[15]	1,634 abstracts	Sentence		●				† Drug combination
ADE [16]	2,972 abstracts	Sentence					●	† Dose relation
BRONCO [17]	108 full texts	Document				●		† Genetic relation
BC5CDR[18]	1,500 abstracts	Document					●	† Induce relation
BioCreative VI PM[19]	5,509 abstracts	Document	●					† Genetic relation
PGxCorpus [20]	945 sentences	Sentence		●		●	●	† Phenotype relation
EMU [21]	110 abstracts	Document				●		† Variant relation<br>‡ Entity spans are not provided
PharmGKB [22]	-	-		●				‡ Repository
DisGeNet [23]	-	-	●					‡ Repository
CTD [24]	-	-		●		●	●	‡ Repository
GWAS [25]	-	-				●		‡ Repository
BindingDB [26]	-	-			●			‡ Repository
BioInfer[27]	1,100 sentences	Sentence	●					×
LLL[28]	167 sentences	Sentence	●					×

IEPA[29]	300 abstracts	Document			●			×
BioCreative II PPI IPS [30]	1,098 full texts	Document	●					×
BioCreative II.5 IPT [31]	122 full texts	Document	●					×
n2c2 2018 ADE[32]	505 summaries	-	●					×
